# Mortality among Alaska Native Adults with Confirmed Hepatitis C Virus Infection Compared with the General Population in Alaska, 1995–2016

**DOI:** 10.1155/2022/2573545

**Published:** 2022-02-08

**Authors:** Sara S. Bressler, Dana Bruden, Leisha D. Nolen, Michael G. Bruce, Lisa Towshend-Bulson, Philip Spradling, Brian J. McMahon

**Affiliations:** ^1^Arctic Investigations Program, Division of Preparedness and Emerging Infections, National Center for Emerging Zoonotic and Infectious Diseases, Centers for Disease Control and Prevention, Anchorage, AK, USA; ^2^Liver Disease and Hepatitis Program, Alaska Native Tribal Health Consortium, Anchorage, AK, USA; ^3^Division of Viral Hepatitis, National Center for HIV, Viral Hepatitis, STD and TB Prevention, Centers for Disease Control, Atlanta, GA, USA

## Abstract

**Background:**

Hepatitis C virus (HCV) infection incidence rates in the United States have increased since 2010 as a byproduct of the opioid crisis despite the introduction of direct-acting antiviral agents in 2013. HCV infection is associated with higher rates of liver-related and nonhepatic causes of death.

**Methods:**

This study compared demographic characteristics and age-adjusted death rates from 1995 to 2016 among Alaska Native (AN) adults infected with HCV (AK-HepC) to rates among the AN and non-AN adult populations living in Alaska. Liver-related disease (LRD) and other disease-specific age-adjusted death rates were compared between the populations.

**Results:**

The all-cause death rate among the AK-HepC cohort was 2.2- and 3.4-fold higher than AN and non-AN adults, respectively, and remained stable over time in all populations. The LRD death rate among the AK-HepC cohort was 18- and 11-fold higher than the non-AN and AN, respectively. The liver cancer rate among the AK-HepC cohort was 26-fold higher compared to the Alaska statewide population. The AK-HepC cohort had elevated rates of death associated with nonhepatic diseases with circulatory disease having the highest rate in all populations. Among liver cancer deaths in the AK-HepC cohort, 32% had HCV listed as a contributing cause of death on the death certificate.

**Conclusions:**

Death rates in the AK-HepC cohort remained stable since 1995 and higher compared to the general population. People with HCV infection had an elevated risk for all-cause, liver-related, and nonhepatic causes of death. Hepatitis C infection may be underrepresented as a cause of mortality in the United States.

## 1. Introduction

Hepatitis C virus (HCV) infection is a blood-borne infection that continues to be a major cause of morbidity and mortality worldwide. In 2013–2016, an estimated 2.4 million people in the United States (US) were infected with HCV, an increase from 2.7 million reported for years 2003–2010 [1]. Most people infected with HCV either are asymptomatic or have mild nonspecific symptoms, while others develop chronic infection and complications, including cirrhosis, liver failure, and hepatocellular carcinoma (HCC), potentially leading to premature death [2, 3]. The overall death rates among those infected with HCV increased during 1999 to 2013 in persons born between 1945 and 1965; an estimated 75% of infected persons are among this baby boomer generation [2, 4, 5]. Direct-acting antiviral agents (DAAs) that provide a high rate of sustained virologic response or cure of chronic HCV infection were introduced in 2013. From 2014 to 2016, HCV-related mortality decreased in the US [6]. Though DAAs have helped reduce the prevalence of chronic HCV infection, the incidence of new HCV infections since 2010 in the US has increased as a byproduct of the opioid crisis [1]. The liver-related mortality in this group may rise in the coming years if this population is not identified by screening and referred for curative treatment.

Hepatitis C-related deaths are likely underestimated. From 2003 to 2013, hepatitis C-related death rates attributable to HCV infection increased in the US, but only 19% of death records from an HCV-infected cohort had HCV infection listed on their death certificate as the underlying or a contributing cause [4, 5]. Hepatitis C-related deaths are not only the result of direct effects of HCV infection on the liver; people with chronic HCV infection are at increased risk of death from other causes [1, 4, 7]. This includes deaths associated with chronic inflammation and inflammatory cytokine release induced by HCV infection which contribute to the development of diabetes, heart disease, and some cancers, as well as related factors that are connected to HCV exposure, such as intravenous drug use (IDU) and mental health disorders [4, 8, 9].

The Alaska Native (AN) population has a higher liver-related death rate compared to other populations in the US [10, 11]. Hepatitis C virus infection likely contributes to this disparity. However, mortality related to HCV infection in AN people has not been examined since 2005, and only liver-related diagnoses were considered. We describe death rates and causes of death among a cohort of AN people with HCV infection in Alaska and compare them to AN and non-AN people in Alaska not infected with HCV.

## 2. Methods

### 2.1. Populations

Alaska Native people make up approximately 20% of the population in Alaska. Many AN persons receive health care through the Alaska Tribal Health System, an integrated tribally operated, statewide, health care system comprised of rural health clinics, regional hospitals, and a tertiary medical center [10, 12]. An AN population-based cohort of persons testing positive for HCV RNA (AK-HepC) has been part of a long-term follow-up study since 1994. The details of recruitment and enrollment of adults have been previously described [10, 12]. The Liver Disease and Hepatitis Program at the Alaska Native Medical Center maintains a registry of all persons chronically infected with HCV for provision of clinical care. Persons from that registry provided consent to take part in a formal study of hepatitis C treatment and liver disease outcomes. This analysis is restricted to adults (≥18 years of age) who consented to be part of the AK-HepC and died during 1995 to 2016. Persons successfully treated were removed from follow-up at the time of their virologic cure, determined by PCR testing for HCV RNA because these people were no longer infected with HCV. Persons who consumed alcohol were not excluded nor were persons with autoimmune liver disease or metabolic syndrome (risks for non-alcoholic fatty liver disease [NAFLD]). For each deceased person in the cohort, the death certificate was pulled to obtain the International Classifications of Disease (ICD) 9^th^ or 10^th^ revision code associated with death. Persons with a liver transplant were counted in the follow-up period after their transplant date. This study was approved by the Alaska Area and the Centers for Disease Control and Prevention Institutional Review Boards and by two Alaska Tribal Health Organizations, the Alaska Native Tribal Health Consortium and Southcentral Foundation.

We compared death rates in the AK-HepC cohort to rates among AN and non-AN people in Alaska. A computerized death certificate database from the Alaska Bureau of Vital Statistics was used to analyze AN and non-Native (non-AN) adult deaths in Alaska from 1995 to 2016 [13]. People in the AK-HepC cohort were removed from the Alaska statewide population. Population estimates for AN and non-AN adults were obtained from the Alaska Department of Labor and Work Force Development and used as a denominator to calculate rates [14].

### 2.2. Statistical Analyses

Demographic analyses and age-adjusted death rates were calculated for each population. Median age at death for the overall Alaska populations was compared to the AK-HepC cohort using the Wilcoxon rank-sum test and gender distribution and year of birth using the likelihood ratio test. Age-adjusted all-cause and liver-related disease (LRD) death rates per 1,000 persons were calculated among adults in all populations from 1995 to 2016 and for time periods 1995–2005 and 2006–2016. Liver-related death rates were defined as having a LRD ICD code, previously described [10, 15], listed anywhere on the death record. Rates were directly adjusted using the 2000 US population as the standard [16]. Rate ratios (RR) and 95% confidence intervals (CI) comparing the death rates among the AK-HepC cohort to the general AN and non-AN adult populations in Alaska were calculated for 1995–2016 and by time period and compared using Poisson regression [17]. Trend over time of all-cause and LRD age-adjusted death rates were analyzed for all populations using Poisson regression.

Attributable causes of death were compared between the populations. The associated causes of death categories were HCV, non-alcohol-related liver disease, alcohol-related liver disease, liver cancer, other hepatitis (including acute hepatitis A, acute hepatitis E, chronic viral hepatitis other than B or C, and unspecified viral hepatitis), nonliver cancer (defined as cancer other than primary liver cancer), HIV, circulatory disease, respiratory disease, diabetes, digestive disease, genitourinary, injury, and mental or behavioral disorders (including disorders due to psychoactive substance use, delusional, mood, and stress-related disorders, behaviors associated with psychological disturbances, adult personality, and mental retardation and disorders of psychological development during childhood and adolescence), and others were previously categorized by ICD 9/10 [4, 15]. Age-adjusted rates for each death category among the AK-HepC cohort were compared to AN and non-AN people in Alaska using the direct method with sufficient sample size. Standardized mortality ratios (SMR) were calculated using the indirect method when the number of deaths for a category was <20 [17, 18]. For each death category, the proportion of deaths that had hepatitis C listed as a contributing cause of death was calculated for each population. Additionally, within the AK-HepC cohort, we calculated the proportion of deaths for each attributable death category from 2006 to 2016 for people born in or before 1965 and compared it to the proportion of people born after 1965. Statistical analyses were done in SAS 9.4, and statistical significance was considered at the *P* < 0.05 level.

## 3. Results

From 1995 to 2016, the AK-HepC cohort comprised 1,051 adult patients 18 years of age or older who provided consent and of whom 280 died during the study period (Table 1). During the same period, there were 15,376 adult deaths in the AN population and 54,757 in the non-AN population living in Alaska. A little over half of the deaths in all three populations were male (AK-HepC cohort: 56.4%, AN population: 55.4%, and non-AN population: 58.5%). The median age at death among the AK-HepC cohort was 53 years (interquartile range: 47–59), which was significantly younger than AN and non-AN persons (median age: 64 and 69 years, respectively, *P* < 0.0001). Of persons who died in the AK-HepC cohort, 77.1% were born between the years 1945 and 1965, which was significantly higher than the deceased AN and non-AN adults in Alaska born in those years (19.9% and 20.0%, respectively, *P* < 0.0001). A larger proportion of AN and non-AN adults were born before 1945 (67.3% and 73.2%, respectively) compared to only 12.5% of adults in the AK-HepC cohort (both *P* < 0.0001).

**Figure 1:**
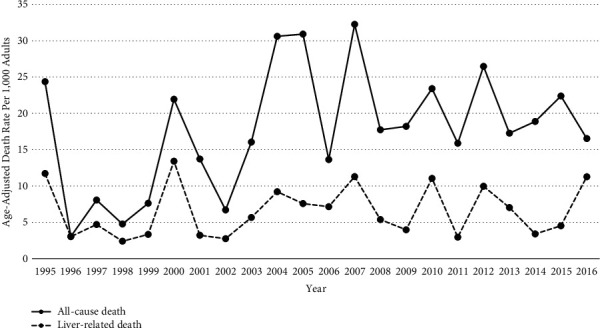
Annual age-adjusted all-cause and liver-related death rates among the Alaska-hepatitis C cohort in Alaska, 1995–2016. ^†^. ^†^Trends are not significant among either group (*P* > 0.05).

The all-cause age-adjusted death rate from 1995 to 2016 among the AK-HepC cohort (19.1 per 1,000 person-years) was significantly higher compared to the AN (11.0, *P* = 0.003) and non-AN populations (7.6, *P* = 0.0005; Table 2). The age-adjusted rate ratios for all-cause death were 2.2 (95% CI: 1.3–3.6) (AK-HepC cohort versus AN adults) and 3.4 (1.7–6.7) (AK-HepC cohort versus non-AN persons living in Alaska). The LRD age-adjusted death rate from 1995 to 2016 among the AK-HepC cohort was 6.8 per 1,000 person-years. This death rate was 18.0 (14.6–22.3) times the rate of non-AN adults and 11.1 (8.9–13.8) times the rate of AN adults. The age-adjusted rate ratios between the AK-HepC cohort and the reference populations did not differ between 1995–2005 and 2006–2016. There was no statistically significant trend in all-cause and liver-related age-adjusted death rates within the AK-HepC cohort over time (Figure 1).

**Figure 2:**
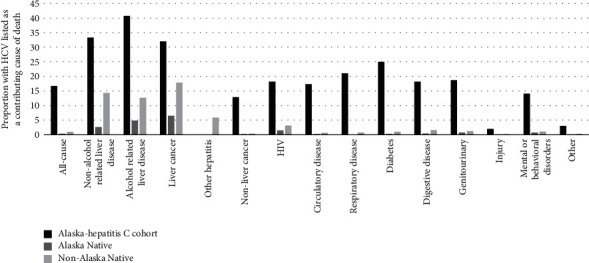
Proportion of death category with hepatitis C virus infection listed as a contributing cause of death among the Alaska-hepatitis C cohort and Alaska Native and Nonnative-Alaskan adults in Alaska, 1995–2016.

Of the 280 AK-HepC cohort deaths, 251 (89.6%) had at least one ICD code listed on their death certificate. This proportion was significantly lower compared to the AN population and non-AN populations where over 99% of deaths had at least one ICD death code (99.5% and 99.6%; *P* < 0.0001, respectively). The age-adjusted rate of HCV infection as a contributing cause of death among the AK-HepC cohort was 2.9/1,000 person-years. This rate was much higher compared to the AN (RR: 71.4 [38.5–132.4]) and non-AN populations (RR: 38.0 [25.0–57.6], Table 3). Among the AK-HepC cohort, 16.7% of known deaths had HCV infection listed as a contributing cause of death (Figure 2). For hepatic-related illness, the AK-HepC cohort had higher death rates associated with non-alcohol-related liver disease (AK-HepC versus AN RR: 9.3 [5.5–15.7], non-AN RR: 15.4 [8.5–28.0]; Table 3), alcohol-related liver disease (AK-HepC versus AN RR: 6.2 [5.0–7.7], AK-HepC versus non-AN RR: 15.1 [9.3–24.4]), liver cancer (AK-HepC versus AN RR: 26.5 [21.0–33.4], non-AN RR: 26.2 [23.8–28.9]), and other hepatitis including acute hepatitis A, acute hepatitis E, chronic viral hepatitis other than B or C, and unspecified viral hepatitis (AN RR: 33.0 [6.8–95.5], non-AN RR: 39.9 [8.2–116.7]) compared to AN and non-AN populations. Among the AK-HepC cohort, the alcohol-related liver disease cause of death category had the largest proportion of deaths with hepatitis C listed as a contributing cause (40.7%) followed by non-alcohol-related liver disease (33.3%) and liver cancer (32.0%; Figure 2). Among AN and non-AN adults in Alaska, liver cancer death category had the largest proportion of deaths with HCV infection listed as a contributing cause (6.5% and 17.9%, respectively).

For nonhepatic conditions, circulatory disease was the highest contributing cause of death for all populations (AK-HepC cohort: age-adjusted rate 5.6, AN: 4.7, non-AN: 3.6) followed by respiratory disease (AK-HepC: 4.7, AN: 2.8, non-AN: 1.6). No significant differences in age-adjusted death rates were found among the AK-HepC cohort and the other populations for circulatory disease; however, for respiratory disease, the AK-HepC cohort had a 2.0 (1.1–3.9) times higher death rate compared to the AN population and 3.7 (1.7–8.0) times higher rate compared to the non-AN population living in Alaska (*P* = 0.03 and *P* = 0.001, respectively). Additionally, the AK-HepC cohort had a statistically higher death rate compared to the Alaska statewide populations for genitourinary diseases (AN RR: 1.8 [1.0–2.9], non-AN RR: 2.8 [1.6–4.6]) while the AK-HepC cohort age-adjusted death rates for diabetes and nonliver cancer were not statistically different compared to the Alaska populations. Among the 31 people in the AK-HepC cohort who died from a nonliver cancer, the top listed cancer was in the lung. Lastly, the AK-HepC cohort showed higher death rates compared to the Alaska statewide populations for death-related mental health or behavioral disorders (AN RR: 2.4 [1.3–4.4], non-AN RR: 5.1 [1.8–14.5]), injury-related deaths (AN RR: 2.0 [1.4–2.7], non-AN RR: 3.7 [2.5–5.5]), and HIV (AN RR: 16.4 [8.2–29.3], non-AN RR: 38.1 [19.0–68.2]).

From 2006 to 2016, there were a total of 146 deaths of people born in or before 1965 and 26 deaths of people born after 1965 in the AK-HepC cohort. Within this cohort, the proportion of deaths with an associated death of cancer other than liver cancer was statistically higher in the group of people born in or before 1965 (16.4% [*n* = 31]) compared to people born after 1965 (0.0% (*n* = 0), *P* = 0.03). The proportion of any other cause of death was not statistically different between people born before 1965 when compared to people born after 1965.

## 4. Discussion

This study examined all-cause and liver-related mortality rates over a 22-year period in AN persons living with chronic HCV infection and compared the rates to AN and non-AN populations living in Alaska. Alaska Native people with HCV infection had an age-adjusted all-cause mortality rate that was 3.4 times higher than the overall rate among non-AN people and 2.2 times higher than the overall AN population. The LRD age-adjusted death rate in the AK-HepC cohort was over 11 times higher than for AN people without known HCV infection and 18 times higher than non-AN people. The AK-HepC population also experienced significantly higher death rates due to liver cancer compared to the AN and non-AN populations.

Hepatitis C virus cohort deaths have been studied in other populations and those deaths occurred at younger ages compared to the reference populations [4, 5, 19, 20]. Results showing elevated death rates in persons living with HCV infection were similar to those found in other prospective HCV cohort studies around the world. A study in Australia showed a seventeen-time higher LRD rate among HCV-infected persons compared to the overall New South Wales, Australian population [9]. Cohorts in Australia and England also had all-cause mortality rates that are higher in HCV-infected people than the overall population [9, 19]. This virus continues to affect the baby-boomer generation but is increasingly impacting younger age groups because of its association with the opioid crisis [1, 19, 20].

Chronic HCV infection has been a problem specifically in the baby-boomer population, people born between 1945 and 1965. Persons with HCV infection in this age group will have likely been infected for more than 30 years, before universal blood safety precautions were advocated, and a larger proportion will have more advanced liver disease [21]. Due to the chronic systemic inflammatory effect of ongoing HCV infection, baby-boomers may have more nonhepatic damage such as atherosclerosis leading to higher rates of coronary artery disease and stroke due to their older age and longer duration of HCV infection than those born after 1965. In contrast, those persons infected more recently during the opioid crisis are less likely to have significant liver damage, due to their shorter duration of infection, and therefore are good candidates for early identification and curative treatment.

People infected with HCV are not only more likely to die of liver cancer, but also more likely to die from other liver- and nonliver-related diseases. This analysis reports a higher liver cancer death rate, similar to other research reporting a 48.6-time higher rate in a group of HCV infected people in the US (The Chronic Hepatitis Cohort Study (CHeCS)) compared to the national rate [4]. This increased rate may be due to the aging population with liver cancer since the antiviral treatments do not stop the formation of tumors in the liver but only slow down the onset [22]. Additionally, given the epidemic of obesity and the metabolic syndrome in the US and Alaska over the last few decades, the copresence of NAFLD could have contributed to the increased rate of HCC. While this analysis did not find a difference in death rates for nonliver cancer, other studies found HCV infected people in the US had higher death rates of kidney, lung, and pancreatic cancers and non-Hodgkin's lymphoma [7]. These findings are similar to the CHeCS group's reporting higher death rates compared to the general population for respiratory, digestive, and genitourinary diseases [4]. These results agree that chronic HCV infection can affect other systems of the body contributing to the higher mortality rates driven by chronic inflammation and inflammatory cytokine release that could contribute to higher rates of diabetes and heart disease [8]. Treatment to cure HCV could likely reduce the higher rates of these nonhepatic complications as we recently observed in a study of improved immunologic function after HCV cure in Alaska [20].

This study also found significantly higher death rates from alcohol-, injury-, HIV-, and mental health-related causes in the AK-HepC cohort compared to other reference groups. Researchers in Australia and the US (CHeCS) found that these associated deaths were listed more frequently as a cause of death among people infected with HCV compared to the general population [4, 9]. This highlights the importance of not only preventing and treating HCV but also recognizing the importance of treating HCV infected people for the conditions that are associated with the acquisition of HCV, through referral to drug addiction and mental health treatment programs.

One concern this study and others have identified is the underreporting of HCV infection as a cause or contributing cause of death on a person's death certificate. Only 16.7% of deaths among the AK-HepC cohort had HCV infection listed as a contributing cause of death. This low percentage is similar to what was found in other HCV cohort studies [4, 9]. In the AK-HepC cohort, even among the liver disease-related deaths and liver cancer deaths, less than 50% had HCV infection listed as a contributing cause on the death certificate. Providers in New York noted the known and unavoidable inaccuracies of overdocumenting non-HCV causes of death. Often, these non-HCV causes of death are documented as immediate reasons minimizing the contribution to mortality due to HCV infection [4]. The multiple medical issues that are seen in the aging baby-boomers may have also led to competing non-HCV-related causes of death including vascular disease and other nonhepatic cancers outweighing deaths due to HCV infection [1].

Our study comes with limitations. This study focused on AN persons with chronic HCV infection which may limit the generalizability of findings. However, the risk associated with acquiring HCV, the genotype distribution found, and overall demographics of this cohort are similar to other US populations infected with HCV [12]. While this study focuses on one population, findings were similar to other US and international populations, suggesting results reflect the overall trends identified in other population studies. Another limitation is the use of death records and ICD death codes. Death records are often not completed by patients' primary physicians and may miss contributing causes of death [5]. The ICD death codes can be unreliable due to the variability in their use. We found that statistically more death certificates from the AK-HepC cohort lacked a cause of death when compared to the general AK population. This limits our ability to evaluate all causes of death. Lastly, the small number of deaths in some death categories did not allow us to calculate RR using the direct method. The magnitude of the ratios is no longer relative to each other since different standard populations were used.

Death rates in this population-based HCV cohort of AN people have not changed since 1995, and the rate differences compared to the Alaska statewide population are similar to other HCV cohorts around the world. Since HCV infection is often asymptomatic for decades and is now disproportionately affecting a younger population, testing based on risk of acquiring HCV infection should be widely undertaken [5, 8]. The DAA therapies have shown remarkable cure rates for those who already have HCV infection; however, drug compliance can be challenging for people with HCV infections associated with the opioid crisis [1]. Programs are ongoing to provide the AN population with easier access to screening and treatment. This study shows how important it is to implement early, aggressive screening to eliminate HCV infection, because eliminating HCV infection can impact death rates not only from LRD, but from a wide range of causes.

## Figures and Tables

**Table 1:** Demographics of deaths among the Alaska-hepatitis C cohort, and Alaska Native and Nonnative people in Alaska, 1995–2016.^†^

Characteristic	Alaska-HepC cohort adults (*N* = 280)	Alaska Native adults (*N* = 15,376)	Alaska-HepC cohort vs. Alaska Native adults	Nonnative-Alaskan adults (*N* = 54,757)	Alaska-HepC cohort vs. Nonnative-Alaskan adults
	N (%)	N (%)	*P* value	N (%)	*P* value
Age in years, median (IQR)	53 (47–59)	64 (47–78)	<0.0001	69 (55–81)	<0.0001
Gender					
Male	158 (56.4)	8,517 (55.4)	0.72	32,024 (58.5)	0.49
Female	122 (43.6)	6,856 (44.6)		22,729 (41.5)	
Age by birth year					
Born before 1945	35 (12.5)	10,348 (67.3)	<0.0001	40,100 (73.2)	<0.0001
Born from 1945 to 1965	216 (77.1)	3,055 (19.9)		10,953 (20.0)	
Born after 1965	29 (10.4)	1,972 (12.8)		3,700 (6.8)	

^†^The Alaska-HepC cohort was removed from the Alaska Native population. Numbers within each category may not add up to total due to missing values.

**Table 2:** Annual age-adjusted death rates for the Alaska-hepatitis C cohort and Alaska Native and Nonnative adults in Alaska, 1995–2016.^†^

Time period	Death rate per 1,000 persons			Age-adjusted rate ratios (95% CI)	
	Alaska-HepC cohort	Alaska Native adults	Nonnative-Alaskan adults	Alaska-HepC cohort vs. Alaska Native adults	Alaska-HepC cohort vs. Nonnative-Alaskan adults
1995–2016					
All-cause deaths	19.1	11.0	7.6	2.2 (1.3–3.6)^*∗*^	3.4 (1.7–6.7)^*∗*^
Liver-related deaths	6.8	0.6	0.4	11.1 (8.9–13.8)^*∗*^	18.0 (14.6–22.3)^*∗*^
1995–2005					
All-cause deaths	16.8	11.1	8.5	2.3 (1.2–4.3)^*∗*^	3.5 (1.5–8.0)^*∗*^
Liver-related deaths	6.8	0.6	0.4	11.7 (6.7–20.3)^*∗*^	20.9 (11.7–37.3)^*∗*^
2006–2016					
All-cause deaths	19.9	11.0	7.1	2.2 (1.4–3.5)^*∗*^	3.4 (1.9–6.2)^*∗*^
Liver-related deaths	7.0	0.6	0.4	10.8 (8.9–13.1)^*∗*^	16.3 (13.0–20.5)^*∗*^

^†^The Alaska HCV cohort was removed from the Alaska Native population. ^*∗*^*P* value<0.05.

**Table 3:** Comparing age-adjusted rate of multiple causes of known deaths between the Alaska-hepatitis C cohort in Alaska and Alaska Native and Nonnative adults in Alaska, 1995–2016.^†^

Death category	Age-adjusted rate per 1,000 adults (crude rate)			Standardized mortality ratio (95% CI)	
	Alaska-HepC cohort	Alaska Native adults	Nonnative-Alaskan adults	Rate ratio/SMR Alaska-HepC cohort vs. Alaska Native adults	Rate ratio/SMR Alaska-HepC cohort vs. Nonnative-Alaskan adults
HCV	2.9 (3.0)	0.03 (0.03)	0.06 (0.06)	71.4 (38.5–132.4)^*∗*^	38.0 (25.0–57.6)^*∗*^
Non-alcohol-related liver disease	3.1 (3.9)	0.4 (0.3)	0.2 (0.2)	9.3 (5.5–15.7)^*∗*^	15.4 (8.5–28.0)^*∗*^
Alcohol-related liver disease	1.7 (1.9)	0.2 (0.2)	0.1 (0.1)	6.2 (5.0–7.7)^*∗*^	15.1 (9.3–24.4)^*∗*^
Liver cancer	2.0 (1.8)	0.08 (0.06)	0.08 (0.07)	26.5 (21.0–33.4)^*∗*^	26.2 (23.8–28.9)^*∗*^
Other hepatitis	−(0.2)	−(0.004)	0.004 (0.004)	33.0 (6.8–95.5)^*∗*^	39.9 (8.2–116.7)^*∗*^
Nonliver cancer	2.0 (2.2)	2.7 (2.0)	2.0 (1.6)	1.0 (0.5–1.9)	1.4 (0.6–3.2)
HIV	−(0.8)	0.04 (0.04)	0.02 (0.02)	16.4 (8.2–29.3)^*∗*^	38.1 (19.0–68.2)^*∗*^
Circulatory disease	5.6 (5.4)	4.7 (3.4)	3.6 (2.8)	1.5 (0.9–2.5)	2.0 (1.0–4.2)
Respiratory disease	4.7 (4.1)	2.8 (2.0)	1.6 (1.2)	2.0 (1.1–3.9)^*∗*^	3.7 (1.7–8.0)^*∗*^
Diabetes	−(0.6)	0.6 (0.4)	0.7 (0.6)	1.4 (0.6–2.7)	1.1 (0.5–2.1)
Digestive disease	−(0.8)	0.7 (0.5)	0.4 (0.3)	1.4 (0.7–2.5)	2.8 (1.4–5.0)^*∗*^
Genitourinary	−(1.1)	0.9 (0.6)	0.6 (0.5)	1.8 (1.0–2.9)^*∗*^	2.8 (1.6–4.6)^*∗*^
Injury	3.4 (3.7)	2.0 (2.0)	1.1 (1.0)	2.0 (1.4–2.7)^*∗*^	3.7 (2.5–5.5)^*∗*^
Mental or behavioral disorders	4.0 (4.6)	2.2 (1.7)	1.2 (1.0)	2.4 (1.3–4.4)^*∗*^	5.1 (1.8–14.5)^*∗*^
Others^‡^	4.4 (4.7)	2.6 (2.4)	1.5 (1.3)	2.1 (1.4–3.1)^*∗*^	3.7 (2.1–6.5)^*∗*^

^†^The Alaska-HepC cohort was removed from the Alaska Native population. Age-adjusted rates were not calculated if <20 deaths. A standardized mortality ratio (SMR) and 95% confidence intervals were calculated using the indirect method when deaths were <20. ^‡^Top listed deaths among other death categories; Alaska-HepC cohort: accidental poisoning by and exposure to narcotics (18.2% of known deaths with other death category, ICD-10: X42), accidental poisoning by and exposure to other and unspecified drugs (15.2%, ICD: X44), accidental poisoning by and exposure to alcohol (12.1%, ICD: X45), anoxic brain damage (9.1%, ICD: G931), and exposure to excessive cold (9.1%, ICD: X31); Alaska Native adults: intentional harm by hanging, strangulation and suffocation (10.3%, ICD: X70), accidental poisoning by and exposure to alcohol (10.0%, ICD: X45), intentional harm-unspecified firearm discharge (9.3%, ICD: X74), sepsis unspecified (7.7%, ICD: A419), and exposure to excessive national cold (7.6%, X31); Nonnative adults: Alzheimer's disease (15.0%, ICD: G309), accidental poisoning by and exposure to other and unspecified drugs (9.6%, ICD: X44), intentional harm-unspecified firearm discharge (8.8%, ICD: X74), injured in motor-vehicle accident (7.7%, ICD: V892), and sepsis, unspecified (6.3%, ICD: A419). ^*∗*^*P* value <0.05.

## Data Availability

Data availability is subject to third party restrictions.
